# Allostatic adaptation and personalized physiological trade-offs in the circadian regulation of the HPA axis: A mathematical modeling approach

**DOI:** 10.1038/s41598-019-47605-7

**Published:** 2019-08-01

**Authors:** Rohit Rao, Ioannis P. Androulakis

**Affiliations:** 1 Department of Chemical and Biochemical Engineering, Rutgers, the State University of New Jersey, Piscataway, USA; 2 Department of Biomedical Engineering, Rutgers, the State University of New Jersey, Piscataway, NJ 08854 USA

**Keywords:** Endocrine system and metabolic diseases, Computational biology and bioinformatics

## Abstract

The hypothalamic-pituitary-adrenal (HPA) axis orchestrates the physiological response to unpredictable acute stressors. Moreover, the HPA axis exhibits prominent circadian activity and synchronizes peripheral circadian clocks to daily environmental cycles, thereby promoting homeostasis. Persistent disruption of homeostatic glucocorticoid circadian rhythmicity due to chronic stress exposure is correlated with the incidence of various pathological conditions including depression, diabetes and cancer. Allostatic habituation of the HPA axis, such that glucocorticoid levels retain homeostatic levels upon chronic exposure to stress, can therefore confer fitness advantages by preventing the sustained dysregulation of glucocorticoid-responsive signaling pathways. However, such allostatic adaptation results in a physiological cost (allostatic load) that might impair the homeostatic stress-responsive and synchronizing functions of the HPA axis. We use mathematical modeling to characterize specific chronic stress-induced allostatic adaptations in the HPA network. We predict the existence of multiple individualized regulatory strategies enabling the maintenance of homeostatic glucocorticoid rhythms, while allowing for flexible HPA response characteristics. We show that this regulatory variability produces a trade-off between the stress-responsive and time-keeping properties of the HPA axis. Finally, allostatic regulatory adaptations are predicted to cause a time-of-day dependent sensitization of the acute stress response and impair the entrainability of the HPA axis.

## Introduction

The hypothalamic-pituitary-adrenal (HPA) axis integrates environmental information and calibrates the internal *milieu*, through an intricate network of glucocorticoid (GC)-sensitive pathways, adapting to predictable and unpredictable environmental variations^1^. The HPA axis is a central player in the response to unpredictable environmental stressors^2^ leading to release of GCs and enabling the host to actively re-establish homeostasis^3^. GCs exhibit circadian rhythmicity, peaking just before the onset of the active phase^4^. Glucocorticoid rhythms enable the host to adjust its behavior in anticipation of periodic changes, including daily variability in light, temperature, food availability and environmental stressors^5,6^. The maintenance of circadian rhythmicity has important functional implications preserving host homeostasis by synchronizing physiology to the environment^7^.

The concept of allostasis was introduced as an extension of the homeostatic framework in order to explain the process by which the host actively maintains physiological stability (homeostasis)^8^. By differentiating between critical homeostatic variables (blood pH, glucose levels etc.), which must be tightly controlled for host survival, and the homeostatic mediators (GCs, norepinephrine) which regulate the former, allostasis attempts to offer a more precise and integrated description of physiological regulation and the stress response^9^. While homeostasis is conventionally based on negative feedback architectures that restore all physiological variables to their basal states in response to stress-induced deviations^10–12^, allostasis describes a labile equilibrium by which the critical homeostatic mediators actively deviate from basal levels to trigger adaptive mechanisms (transiently altered activity of relevant metabolic, immune and stress-responsive signaling pathways) that enable the host to cope with perceived stressors and subsequently re-establish homeostasis followed by their gradual return to pre-stress levels^13,14^.

However, chronic deviation of glucocorticoids from their homeostatic levels through prolonged activation results in a physiological cost (allostatic load) and is associated with detrimental outcomes^15,16^. Habituation of the HPA axis, such that GCs return to or maintain homeostatic levels in response to chronic exposure to the same stressor provides the host with fitness advantages by optimizing energetic resources and preventing the persistent dysregulation of downstream GC-responsive signaling mechanisms^17–19^. However, successful habituation to chronic stress requires the activation of further adaptive allostatic regulatory mechanisms, which can alter the homeostatic functioning of related physiological systems when the host is exposed to novel environments or stressors. Habituation to chronic stress does not necessarily imply an attenuation of allostatic load, and failure of such attenuation results in long-term disruptions, further compromising homeostasis.

Considering the practical difficulty in measuring changes in internal physiological state (e.g. change in energetic expenditure and intake) in a variable environment^8,20^, it is suggested that monitoring changes in the concentrations of the homeostatic mediators such as GCs can be indicative of the allostatic state of the host. However, in the case of a habituating allostatic adaptation to chronic stress, when little discernible change in the levels of glucocorticoids occurs, it becomes increasingly difficult to characterize the allostatic state of the system. Moreover, the observation of apparently nominal levels of glucocorticoids upon chronic stress habituation might conceal underlying regulatory changes in the HPA axis that could impair its homeostatic functioning. In such cases, a modeling-based approach can be a particularly useful tool for generating and evaluating experimentally verifiable hypotheses^21^.

We proposed a semi-mechanistic mathematical HPA model predicting functionally relevant adaptations that occur upon habituation to chronic stress. Amongst the primary functions of the HPA axis are: a) its role as the major physiological stress response system; and b) its ability to synchronize (or entrain) critical internal physiological processes to periodically varying external environment cues (zeitgebers). Therefore, in this work we highlight chronic stress-induced allostatic adaptations influencing its stress-responsiveness and entrainment properties, individual differences in susceptibility to allostatic load, and the existence of multiple individualized regulatory strategies enabling the host to conserve a basal circadian phenotype while allowing for flexibility of its stress-responsive and entrainment properties. We demonstrate that this regulatory diversity results in trade-offs between the stress-responsiveness of the HPA axis and its time-keeping ability. An improved understanding of the underlying regulatory architectures leading to allostatic adaptations and functional trade-offs can provide insights into the complex physiological signaling of the HPA axis in health and disease.

## Methods

### HPA axis model

The primary mediators of the HPA axis are represented by nonlinear ODEs that form a limit-cycle type Goodwin oscillator^22^. The Goodwin oscillator represents a phenomenological prototypical biological oscillator, initially devised to show the possibility of self-sustained oscillations in a genetic circuit with a simple delayed negative feedback loop architecture. The skeletal structure of the Goodwin oscillator adapted to model the circadian oscillations in the HPA axis was initially devised by Sriram *et al*. and was modified by our group to account for the entrainment of the HPA axis rhythms by light via the SCN in both nocturnal and diurnal species.

Briefly, according to the scheme of equations presented here, CRH induces the release of ACTH, which subsequently induces the release of CORT (corticosterone for the purpose of our study) (Equation 7–9). While the model can be calibrated to glucocorticoid circadian rhythms in both diurnal and nocturnal species, in the subsequent section we calibrate the model to experimental CORT circadian rhythms from female Lewis rats in the proestrous phase of the estrous cycle, when estrogen levels are at their highest^23^.

The synthesis of CRH is described by zero-order kinetics (${K}_{p1}$), while the synthesis of ACTH and CORT is described by first-order kinetics (${K}_{p2}$, ${k}_{p3}$, respectively). The degradation of each of the three mediators is described by Michaelis-Menten kinetics. The use of Michaelian kinetics in our model scheme precludes the use of unreasonably high values of the Hill coefficient^24,25^, generally interpreted as the number of ligand subunits cooperatively binding to a receptor. Moreover, CRH, ACTH and the glucocorticoids all undergo enzymatic metabolism or degradation^26–28^. We further account for glucocorticoid receptor (GR)-mediated pharmacodynamics as adapted from Ramakrishnan *et al*.^29^ (Equation 10–13). The binding of released CORT to cytosolic GR, modeled using second-order kinetics (Equation 11), results in the formation of the receptor-glucocorticoid complex (DR). DR(N) represents the nuclear activated receptor-glucocorticoid complex assumed to be responsible for the receptor-mediated effects of glucocorticoids. The receptor mediated inhibition of CRH and ACTH by the glucocorticoids (through DR(N)) is described in Equations 7 and 8, respectively and accounts for the negative feedback loop of the HPA axis. ${K}_{p1}$ and ${K}_{p2}$ are the inhibition constants and are indicative of the strength of inhibition in the negative feedback loop, with smaller values indicative of stronger negative feedback. Furthermore, Equation 10 describes the negative regulation of DR(N) on the transcription of GR mRNA.

Furthermore, we account for the entraining influence of light on the HPA axis. Kalsbeek *et al*. argue that light has an inhibitory influence on CRH production in nocturnal animals^30^. Accordingly, given that we calibrate our model to CORT data form nocturnal animals, we assume that light mediates its inhibitory influence by inducing the degradation of CRH in our model. Furthermore, we account for a 1–2 hour delay between the start of light exposure and the onset of the photo-induced effects on the HPA axis (denoted by the term $ligh{t}_{effect}$)^31^. While using a step function to model the light exposure, we use a Hill function to model the ultrasensitive response of the phototransduction pathways (Equation 1–6). Detailed descriptions of the parameters are provided in the Supplementary Materials and Methods.

*HPA axis mediators*

1 $$light=\{\begin{array}{c}1\,7:00\le t\le 21:00\\ 0\,21:00 < t < 7:00\end{array}$$

2 $$\frac{dligh{t}_{TCsynth1}}{dt}={k}_{t}(light-ligh{t}_{TCsynth1})$$

3 $$\frac{dligh{t}_{TCsynth1i}}{dt}={k}_{t}(ligh{t}_{TCsynthi-1}-ligh{t}_{TCsynthi}),\,i=3$$

4 $$\frac{dligh{t}_{TCdeg1}}{dt}={k}_{t}(ligh{t}_{deg}-ligh{t}_{TCdeg1})$$

5 $$\frac{dligh{t}_{TCdegi}}{dt}={k}_{t}(ligh{t}_{TCdegi-1}-ligh{t}_{TCdegi});i=3$$

6 $$\frac{dligh{t}_{effect}}{dt}={k}_{us}\frac{ligh{t}_{TCsynth\,i}^{n}}{{K}_{M,us}^{n}+ligh{t}_{TCsynth\,i}^{n}}-{k}_{deg,us}ligh{t}_{effect}(1+{k}_{eff}ligh{t}_{TCdegi})$$

7 $$\frac{dCRH}{dt}=\frac{{k}_{p1}.{K}_{p1}}{{K}_{p1}+DR(N)}-{V}_{d1}.\frac{CRH\cdot (1+\frac{ligh{t}_{effect}}{1+ligh{t}_{effect}})}{{K}_{d1}+CRH}$$

8 $$\frac{dACTH}{dt}=\frac{{k}_{p2}.{K}_{p2}CRH}{{K}_{p2}+DR(N)}-{V}_{d2}.\frac{ACTH}{{K}_{d2}+ACTH}$$

9 $$\frac{dCORT}{dt}={k}_{p3}.ACTH-{V}_{d3}.\frac{CORT}{{K}_{d3}+CORT}$$

10 $$\frac{dG{R}_{mRNA}}{dt}={k}_{sy{n}_{GRm}}.(1-\frac{DR(N)}{I{C}_{{50}_{GRm}}+DR(N)})-{k}_{deg}.G{R}_{mRNA}$$

*Glucocorticoid receptor pharmacodynamics*

11 $$\frac{dGR}{dt}={k}_{syn,GR}.G{R}_{mRNA}+{r}_{f}.{k}_{re}.DR(N)-{k}_{on}.(CORT).GR-{k}_{deg,GR}.GR$$

12 $$\frac{dDR}{dt}={k}_{on}.(CORT).GR-{k}_{T}.DR$$

13 $$\frac{dDR(N)}{dt}={k}_{T}.DR-{r}_{f}.{k}_{re}.DR(N)$$

### Model calibration to experimental data

The model equations result in the generation of endogenous oscillations given an appropriate choice of parameters. The model was calibrated to experimental data obtained by Atkinson and Wadell on CORT circadian rhythms in female Lewis rats during the proestrous phase of the estrous cycle^23^. Atkinson and Wadell performed cosinor rhythmometry analysis, a commonly used technique in circadian research, to determine the circadian parameters of CORT data. In calibrating our model, we match a cosinor fitted to our simulated data to the experimental cosinor profile. Further details regarding the calibration of the model to experimental data are provided in the Supplementary Materials and Methods. Since, rats in the experimental study were exposed to a 14-hour light/10-hour dark photoperiod we use an identical light schedule to entrain the HPA axis in our model.

**Figure 1:**
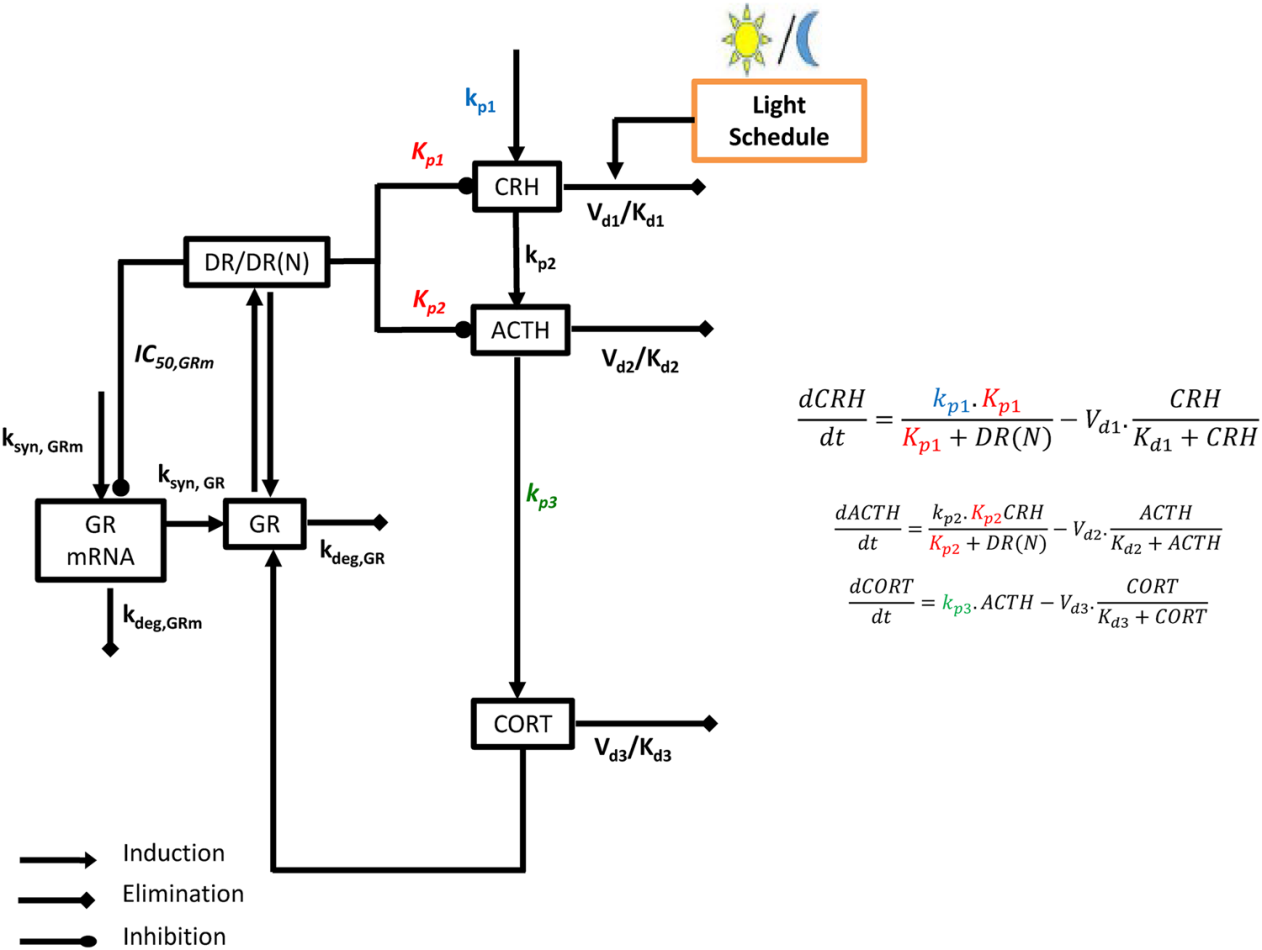
Schematic depiction of the modeled HPA axis network. The feedforward adrenal sensitivity, (k_p3_; green) and the hypothalamic negative feedback (K_p1_; red) and pituitary negative feedback (K_p2_; red) account for regulatory variability in the system. The value of the zero-order rate constant for CRH synthesis (k_p1_) (blue) is increased to simulate chronic stress by increasing the central hypothalamic drive to the HPA axis. The model equations that account for these parameters are shown on the right.

Host homeostatic mechanisms maintain the circadian rhythms of glucocorticoids, the primary effector molecules of the HPA axis, within strict physiological bounds. However, within these physiological bounds glucocorticoid rhythms exhibit substantial inter-individual as well as intra-individual variability. Thus, we subsequently accounted for this homeostatic variability in the regulatory processes of the HPA axis in our mathematical model. In doing so, we hypothesize that differences in the negative feedback (defined by parameters ${K}_{p1}$ and ${K}_{p2}$ in our model) and feedforward adrenal sensitivity (${k}_{p3}$) within the HPA axis define the regulatory variability in its homeostatic activity while also adjusting in a compensatory manner to conserve the CORT circadian phenotype within reasonable physiological bounds. While regulatory variability could be accounted for by using more parameters, for simplicity we limited to our analysis to the above three parameters, which represent physiologically important feedforward as well as feedback processes in the HPA axis. A schematic of the mathematical model is shown in Fig. 1, with the processes accounting for regulatory variability in the system highlighted in red and green. A sampling-based approach was adopted to characterize the homeostatic variability in the HPA axis such that the circadian parameters of the simulated profiles lie within ±1 standard deviation of the experimentally obtained circadian parameters for the CORT profiles in^23^ from the cosinor rhythmometry analysis. Furthermore, rather than inferring system properties from a single optimal parameter set, such an approach allows us to identify subspaces of the three regulatory parameters (${K}_{p1}$, ${K}_{p2}$ and ${k}_{p3}$) that correspond to homeostatic glucocorticoid circadian rhythms. Details regarding the sampling methods are given in the Supplementary Materials and Methods and in our previous work^32^. It should be noted that we are primarily concerned with studying the qualitative behavior of glucocorticoid circadian dynamics in this work and have not simultaneously calibrated our model to experimentally observed circadian rhythms of CRH and ACTH. As a result, a limitation of our model is that the circadian dynamics of CRH and ACTH are outside the dynamic range that is physiologically observed for these hormones. While such a limitation has been observed for a number of other published mathematical models^33,34^ of the HPA axis, we expect that the key qualitative relationships described by our subsequent simulations will be maintained, should future models more quantitatively capture the dynamics of the other HPA axis mediators.

### Determining regulatory adaptations to chronic stress

While transient HPA axis activation is associated with adaptive benefits, excessive glucocorticoid secretion results in the disruption of nominal circadian rhythms and pathophysiological changes in downstream glucocorticoid-sensitive signaling systems. Therefore, to prevent persistent dysregulation, the host habituates to an unresolved low-level chronic stressor through adaptations in regulatory mechanisms thus, enabling the maintenance of homeostatic glucocorticoid circadian rhythmicity. We aimed to characterize these putative regulatory adaptations of the system upon habituation to chronic stress exposure. Experiments show that chronic stress can have complex effects on the properties of the HPA axis^34^. We simplify the effects of chronic stress on the HPA axis by lumping its influence in a single term, the zero-order rate constant for CRH synthesis, ${k}_{p1}$, representative of the central activation of the HPA axis. We simulated a chronic stress regimen by adopting a method used by Sriram *et al*.^35^ whereby ${k}_{p1}$ is increased to a chronically elevated value, thus simulating an increase in central CRH drive to the HPA axis as a result of the chronic stress. Following this, we resample the three parameters, ${K}_{p1}$, ${K}_{p2}$ and ${k}_{p3}$, which define the nominal parametric subspace, such that the corticosterone profiles generated by the resampled parameters in the chronically stressed state also satisfy the error criteria with respect to the homeostatic experimental corticosterone profiles. Chronic stress is known to influence glucocorticoid receptors^36^, inducing glucocorticoid feedback resistance; influence glucocorticoid metabolism^37^, pituitary ACTH dynamics^38,39^ etc. However, for conceptual and computational simplicity, we assume that only the three parameters hypothesized to account for regulatory variability, representing important feedback and feedforward processed, are involved in allostatic adaptation. This procedure was carried out for two different values of ${k}_{p1}$ (highlighted in blue in Fig. 1), representing an “intermediate” and “high” level of chronic stress.

Following the simulated exposure to chronic stress, we determine the possible alterations in the acute stress response of the system. The simulated acute stressor results in a transient increase in the rate of CRH synthesis. This transient increase in CRH synthesis is a simplified general representation that might simulate the effect of various acute stressors, both psychological, such as restraint stress as well as physiological, such as bacterial lipopolysaccharide (LPS) exposure. The CORT response to the acute stressor is characterized by the difference in area under the curve (AUC) between the respective CORT profiles with and without exposure to the acute stressor for 4 hours from the time of in-silico exposure to the acute stressor. Subsequently, we used a method of symbolic representation developed by Lin *et al*.^40^ to partition the acute stress response in the nominal condition and the two chronic stress conditions in order to more closely study individual variability in the acute stress response within each parametric space and how this variability might be altered after allostatic adaptation. Briefly, the acute stress response for each of the parameter sets comprising the three surfaces was pooled together, and z-scored. Following this, the z-scored values were partitioned into five equiprobable regions, and were assigned symbols from 1–5, with 1 and 5 representing the z-scored values at the lower and upper tails of the normal distribution, respectively. For a more detailed description of the procedure the reader is referred to original publication by Lin *et al*.^40^.

### Determining the influence of individual variability and chronic stress on the flexibility of entrainment

Appropriate temporal adaptation or entrainment of critical physiological systems, such as the HPA axis, to the daily rhythmic variations in the environment^41^, such as periodic photic and temperature cues (zeitgebers), is fundamental to host survival. We hypothesized that chronic stress habituation of the HPA axis would alter the flexibility of the system to entrain to externally varying zeitgebers. We aimed to characterize both individual differences in the entrainment properties (differences in properties within each parametric subspace) as well as the differences in entrainment properties occurring as a result of the adaptation to the simulated chronic stress regimen (differences in properties between the three parametric subspaces). A standard metric for evaluating the entrainment characteristics of the endogenous oscillator is to assess the range of entrainer periods for which the endogenous oscillator is phase-locked to the entrainer as a function of the coupling strength between them^42^. The domain of entrainment, referred to as the Arnold tongue, is indicative of how flexibly the endogenous circadian oscillator is entrained to changes in the period of the environmental zeitgeber^43,44^. While flexibility in entrainment characteristics might enable the system to adequately adapt to changes in external zeitgebers, over-responsiveness on the other hand, might compromise the time-keeping properties of the oscillator by making it too sensitive to noisy environmental stimuli. In determining the possible parametric dependence of the entrainment properties, we calculated the Arnold Tongue for nine representative parameter sets on each of the three parametric surfaces. The nine points on each surface were selected in sets of three such that each set of three had approximately the same level of adrenal sensitivity (k_p3_) and such that they span the parametric subspaces accounting for regulatory variability. Specific details on the determination of the Arnold tongues are provided in the Supplementary Materials and Methods.

### Response to transient and permanent changes in the light-dark schedule

Since photoperiod is the primary entrainer of internal circadian rhythms, we determined the parametric dependence of the ability of the system to respond to two physiologically relevant perturbations in the light-dark schedule. In the first case, we studied the response of the system to a transient (96-hr) inversion in the light schedule, simulating temporary shift-work. During this protocol, the maximum phase difference between nominal profiles and those exposed to transiently inverted light schedule was measured and was used to characterize the extent of phase sensitivity to shift work. Subsequently, we determined the parametric dependence of the ability of the system to re-synchronize to a permanent, abrupt shift in the light/dark schedule, simulating the response to jet-lag. On the day of the simulated permanent shift, the time of lights ON was delayed by 10-hr to 17:00, while maintaining the same photoperiod of the light/dark cycle subsequent to the day of the shift. Profiles were considered to be re-synchronized if the phase difference between two consecutive CORT peaks was within 3 minutes.

### Determining the influence of individual variability and chronic stress on the robustness of HPA axis oscillations

In addition to the ability of the HPA axis to flexibly entrain to environmental zeitgebers, it must also remain robust to transient perturbations in its rhythm, and swiftly relax to its nominal rhythmic activity after mounting an adequate response to an acute stressor. For a periodic system, this property is quantified by the Floquet exponent, which is representative of the system’s stability to amplitude perturbations^45^. Greater (stable) Floquet exponents (in absolute value) imply the system is able to robustly restore its homeostatic rhythm (relaxation) after a transient perturbation from its homeostatic oscillatory state. The procedure for the calculation of the Floquet exponents is described in the Supplementary Materials and Methods.

## Results

### Homeostatic diversity of regulatory mechanisms

In the context of allostasis, substantial flexibility in glucocorticoid responsiveness enables the host to adequately adapt to acute environmental challenges^9,20^. However, given the central regulatory importance of glucocorticoids there is, at the same time, an evolutionary advantage for the host to conserve basal glucocorticoid activity within reasonable physiological bounds such that compensatory downstream homeostatic processes necessary for host survival are optimally balanced^8^. Thus, we aim to show computationally that the physiological need for the system to conserve a primary phenotype, i.e., basal circadian activity, while at the same time accounting for adequate reactiveness, permits the existence of multiple individually “customized” regulatory strategies in order to achieve this goal^46^.

**Figure 2:**
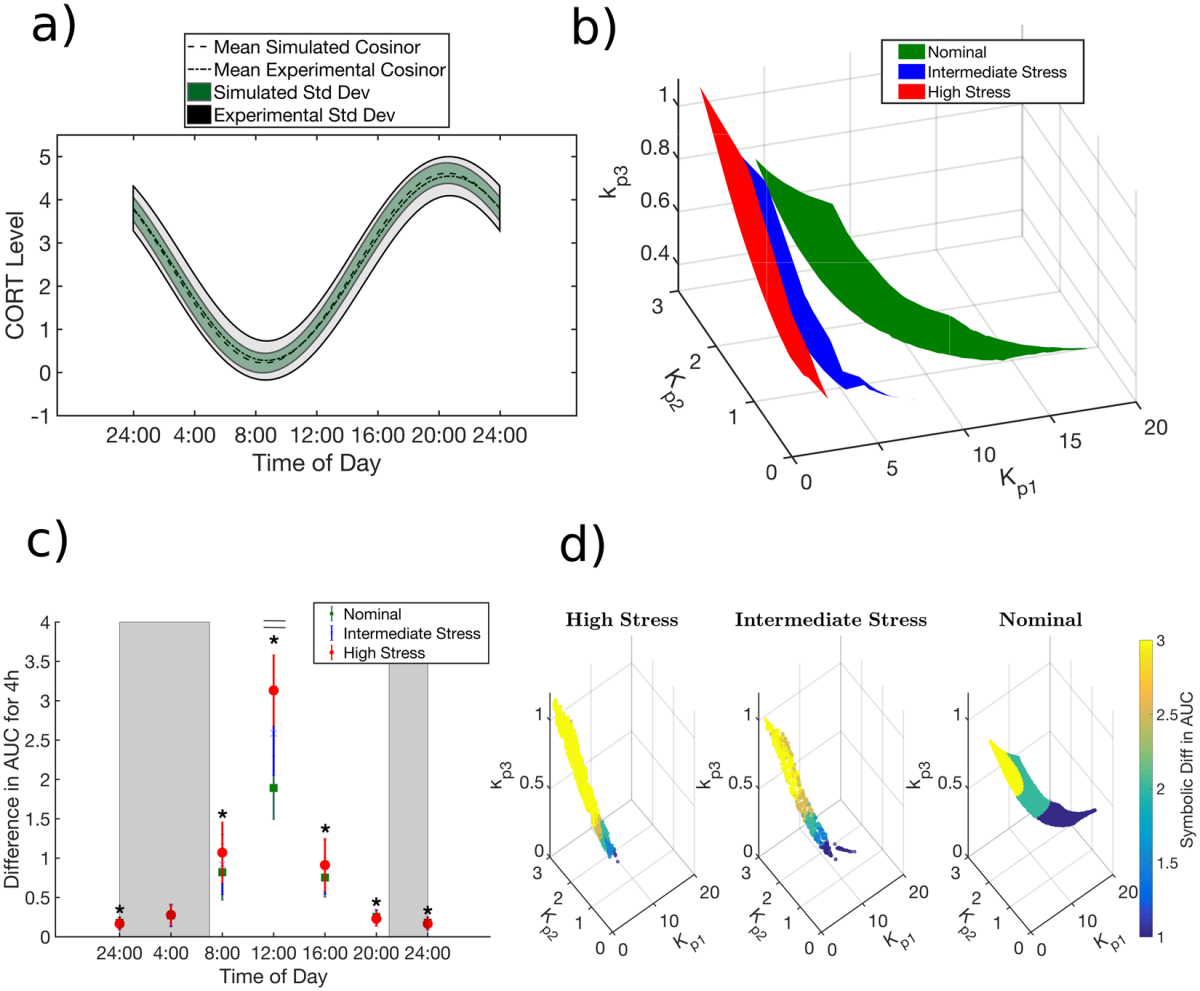
(**a**) Simulated corticosterone cosinor rhythms that qualitatively match experimentally obtained cosinor rhythms as generated by the parameter sets constituting the (**b**) nominal parameter space (green). There is a balance between the three regulatory mechanisms considered here such that they match the experimental rhythms. Upon habituation to chronic stress the nominal parameter space (green to blue and red) allostatically adapts to increasing levels of chronic stress such that the system still produces nominal corticosterone rhythms. The allostatic adaptations include a substantial increase in the average strength of hypothalamic negative feedback decrease (sharp decrease in the range of the K_p1_ values that the system can take). Furthermore, there is an increase in the average adrenal sensitivity (k_p3_) and a slight decrease in the strength of pituitary negative feedback (K_p2_). These adaptations result in a decrease in the area of the surfaces with increasing levels of chronic stress, which implies a decrease in underlying regulatory flexibility of the system. The area of the nominal parametric subspace is 0.7702 a.u. (arbitrary units), that of the intermediate stress parametric subspace is 0.5108 a.u., and that of the high stress parametric subspace is 0.2216 a.u. (**c**) The acute stress response of the system is increased when exposed to a stressor in the middle of the inactive phase. The response at this time of day is substantially sensitized after allostatic habituation to chronic stress.  (**d**) In both the nominal and chronically stressed conditions, the acute stress response increases with increasing adrenal sensitivity. Moreover, we find that there is a sensitization of the stress response (in the middle of the inactive phase) upon habituation to chronic stress. At the same level of adrenal sensitivity, chronically stressed individuals respond more strongly to a subsequent stressor than individuals in the nominal case. Furthermore, individuals with higher adrenal sensitivity exhibited greater stress sensitization as indicated by the increase in the proportion of parameters exhibiting the most robust acute stress response (denoted by the yellow region).

In characterizing the underlying regulatory variability in the system, we hypothesize that the primary regulatory processes adjusting to conserve the circadian phenotype involve: a) the feed-forward adrenal sensitivity (${k}_{p3}$); and b) the negative feedback effects of receptor-bound glucocorticoids on the hypothalamus (the strength of hypothalamic negative feedback indicated by ${K}_{p1}$), inhibiting CRH release, and on the pituitary (the strength of pituitary negative feedback is indicated by ${K}_{p2}$), inhibiting ACTH release. Furthermore, the model accounts for the synchronizing influence of light-mediated signaling on the secretion of CRH. We calibrate our model to CORT circadian profiles in female Lewis rats in the proestrous phase of the estrous cycle as obtained from experiments by Atkinson and Waddell^23^. We used a light-schedule identical to the one adopted in this study in order to keep our simulations consistent with experimental conditions. Thus, given an appropriate selection of parameters the light-entrained model of the HPA axis generates CORT circadian profiles that qualitatively match the experimentally obtained CORT circadian profiles. A parametric analysis of our model indicates that homeostatic glucocorticoid rhythms^23,32^ (Fig. 2a) are attained within a well-defined region of parameter values of the three key regulatory arms (Fig. 2b). We hypothesize that the parameter sets constituting this parametric subspace are indicative of individual variability in the regulatory constitution of the system. The results of Fig. 2b indicate that the adrenal responsiveness and negative feedback processes vary in an interdependent manner, such that changes in adrenal sensitivity (${k}_{p3}$) are compensated for by adjustments in the strength of negative feedback (${K}_{p1}$) in order to maintain the homeostatic circadian patterns of glucocorticoid activity. Therefore, our results emphasize that the feedforward and feedback processes of the HPA network can be systematically tuned, and adapted, such that they conserve the circadian phenotype, establishing the regulatory diversity of homeostasis.

### Allostatic adaptation to chronic stress

Allostatic habituation to an unresolved low-level chronic stressor enables the host maintain pre-stress glucocorticoid rhythms, preventing the potentially pathological disruption of GC-sensitive signaling pathways. However, these adaptations are associated with a physiological cost, likely the result of allostatic load accumulation^47^. Thus, once establishing the regulatory homeostatic diversity, we aimed to characterize habituating allostatic regulatory adaptations to chronic stressors as well as possible functional changes associated with such allostatic adaptations. As proposed earlier^35^, chronic stress is assumed to be the result of an over-activated hypothalamus (represented in our model by persistently increased values of ${k}_{p1}$). Allostatic habituation to chronic stress leads to the adaptation of regulatory arms in order to maintain the circadian levels of glucocorticoids. Specifically, the model predicts that allostatic habituation as a result of a chronic increase in hypothalamic drive to the HPA axis primarily causes an increase in the hypothalamic negative feedback (decreased ${K}_{p1}$ levels) along with a reduction in the range of attainable ${K}_{p1}$ (Fig. 2b) and an average increase in the adrenal sensitivity, ${k}_{p3}\,$of the system. These changes were also accompanied by a slight decrease in the average pituitary negative feedback (increased ${K}_{p2}$ levels). Regulatory diversity is still observed upon allostatic habituation, as indicated by the fact that an ensemble of regulatory parameters maintains homeostatic rhythms, however, an overall loss in the regulatory variability of the system is also observed, as indicated by the reduction in the overall area of the parametric subspaces as the level of chronic stress increased. In other words, the system still maintains its regulatory diversity even under conditions of chronic stress and allostatic habituation. However, the regulatory flexibility of the system is reduced thereby, rendering it more difficult to maintain homeostatic rhythms, indicative of allostatic load accumulation.

If the system, despite chronic stress and the associated allostatic load, is still able to maintain its homeostatic phenotype, it is natural to question whether the regulatory adaptations compromise the functional characteristics of the system. In order to better understand and quantify the implications of allostatic load and the associated regulatory adaptations in terms of the functional characteristics of system, we analyzed the system’s response to an acute stressor subsequent to allostatic habituation. The acute stressor is simulated by inducing a transient increase in CRH production, as a surrogate to an acute HPA axis stimulant^48^. The HPA response to acute stress is quantified by calculating the area under the curve (AUC) of CORT over a 4 hr period following exposure to the (acute) stress^49^. We determine that habituation to chronic stress results in time-of-day dependent alterations in the system’s acute stress response. Specifically, upon habituation to chronic stress, the acute stress response appears to become more robust if experienced towards the middle of the inactive phase relative to the nominal homeostatic state (Fig. 2c). In studying the individual differences in acute stress response we used a method of symbolic representation developed by Lin *et al*. to semi-quantitatively partition the acute stress response within each parametric subspace. This symbolic representation of the difference in AUC, referred to as the symbolic difference in AUC, is depicted in Fig. 2d. In the nominal case without exposure to chronic stress, we determined that individuals with higher adrenal sensitivity tend to have a stronger (larger yellow region) acute stress response (Fig. 2d). We hypothesized that chronic stress habituation would alter these individual differences in acute stress response during the inactive phase. We determine that chronic stress habituation results in a sensitization of the acute stress response in the inactive phase, such that subjects with the same adrenal sensitivity respond more strongly in the chronically stressed state (Fig. 2d). Therefore, the sensitization of the acute stress response potentially subjects downstream glucocorticoid-sensitive signaling pathways to pathological glucocorticoid overexposure. This phenomenon of chronic stress sensitization has been frequently observed experimentally, where habituation to a chronic stressor results in a more pronounced response to an acute stressor in comparison to the homeostatic state^50^.

### Intrinsic ability of adaptation *versus* relaxation

**Figure 3:**
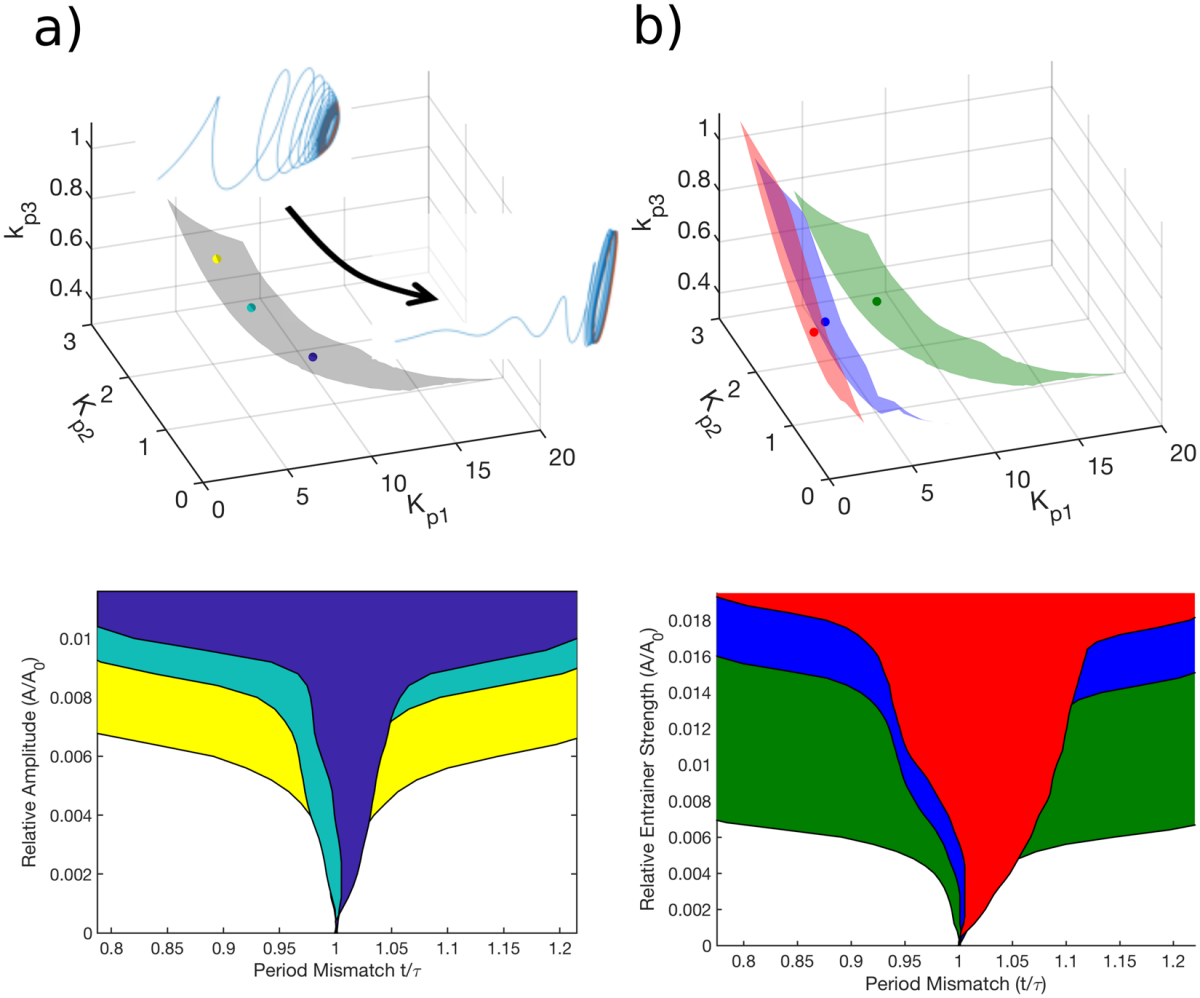
The appropriate entrainment of the HPA axis to changes in the environmental zeitgeber is critical to host survival. The domain of entrainment (depicted by the Arnold tongue) is indicative of the responsiveness of the endogenous circadian oscillator is to frequency fluctuations in a zeitgeber. (**a**) The domain of entrainment decreases with decreasing adrenal sensitivity (k_p3_). The parameter set with highest adrenal sensitivity is depicted in yellow, that with intermediate adrenal sensitivity is depicted in cyan and that with the lowest adrenal sensitivity is depicted in dark blue (Top). The domains of entrainment (Arnold tongues) are shown for the three representative parameter sets on the nominal parametric surface with decreasing adrenal sensitivity (Bottom). This implies that simulated individuals with higher adrenal sensitivity are more flexibly entrained to changes in the zeitgeber frequency. Parameter t represents the entrainer period, while τ represents the intrinsic period of the oscillator. *A* represents the amplitude of the entrainer, while *A*_*0*_ represents the amplitude of the oscillator. Furthermore, we find that the stability of the system to amplitude perturbations increases with decreasing adrenal sensitivity as depicted qualitatively by the rapid return of the system to the stable homeostatic oscillatory solution after an acute perturbation (Top). (**b**) The domain of entrainment decreases with increasing levels of chronic stress for parameter sets at the same level of adrenal sensitivity. The parameter set in the nominal unstressed case is depicted in green, the parameter set in the case of intermediate stress is depicted in blue, and that in the case of high stress is depicted in red (Top). The decrease in the domain of entrainment with increasing levels of chronic stress when comparing parameter sets at the same level of adrenal sensitivity (Bottom) implies that chronic stress decreases the ability of the system to be flexibly adapt to changes in the zeitgeber frequency. t represents the entrainer period, τ represents the intrinsic period of the oscillator, *A* represents the amplitude of the entrainer, while *A*_*0*_ represents the amplitude of the oscillator. The relative amplitude is indicative of the entrainer strength.

The ability of rhythmic endogenous systems, such as the HPA axis, to synchronize to environmental, as well as internal zeitgebers is of critical evolutionary and adaptive significance^51^. Our earlier results demonstrated that under the entraining influence of light, the parametric surfaces, shown in Fig. 2b, indicative of underlying regulatory diversity, generate homeostatic glucocorticoid circadian rhythms. We hypothesized that this regulatory diversity will influence the intrinsic ability of the system to flexibly adapt to changes in zeitgeber characteristics. Therefore, as a measure of the adaptability of the system, we aim to determine whether the ability of the endogenous oscillator to be entrained by a zeitgeber is influenced as a result of either regulatory diversity or regulatory adaptations to chronic stress exposure. Interestingly, our model predicts that the higher the adrenal sensitivity the larger the domain of entrainment (Fig. 3a, top and bottom panel) (greater flexibility in adaptation to changing environments). Moreover, the regulatory adaptations associated with habituation to chronic stress result in a reduced the domain of entrainment (Fig. 3b, top and bottom panel). Thus, the observed allostatic habituation makes it harder for simulated individuals to adapt to fluctuations in zeitgber frequencies, despite having maintained homeostatic levels of HPA output, implying that in order to maintain the same adrenal sensitivity, the system must adjust its regulatory processes such that it compromises on its ability to adapt to zeitgeber fluctuations. A related question concerns the relation between the underlying regulatory characteristics of an oscillatory system and its ability to robustly restore its homeostatic rhythm (relaxation) after a transient perturbation from its homeostatic state. This is characterized by calculating the Floquet exponents of the system (see Methods and Supplementary Methods) and is indicative of the stability of the system to amplitude perturbation, or in other words the robustness of its oscillatory characteristics. We performed multiple linear regression analysis to determine the dependence of the relaxation characteristics (given by the leading Floquet exponent) of the oscillator on the three regulatory parameters of interest (${K}_{p1}$, ${K}_{p2}$ and ${k}_{p3}$) in our system. Our analysis indicates that individuals characterized by a larger domain of entrainment, i.e., more easily entrained, also exhibit lower glucocorticoid oscillation amplitudes, and tend to be characterized by slower rates of relaxation (smaller absolute value of leading Floquet exponents) thus, indicative of a slower rate at which the system returns to homeostasis following an acute perturbation (Fig. 3a and Supplementary Figs S2 and S3). In other words, we predict that the more easily the system is entrained, the more sensitive it becomes to acute perturbations from its homeostatic rhythm. Abraham *et al*.^44^ show both theoretically using limit cycle oscillators of varying complexity as well as experimentally for circadian oscillators in the suprachiasmatic nucleus (SCN) and in fibroblasts that for a given zeitgeber strength the range of entrainment of these oscillators varies inversely with their intrinsic amplitude and the rigidity of the oscillator given by its amplitude relaxation rate (inferred from the Floquet exponent). Thus, an oscillator with a higher amplitude, termed a “strong” oscillator has a narrower range of entrainment and is more rigid (higher Floquet exponent) compared to a “weak”, low amplitude oscillator. Multilinear regression analysis shows that the calculated leading Floquet exponents are strongly influenced by the adrenal sensitivity of the system and decrease with increasing adrenal sensitivity for nominal as well as chronically stressed parametric subspaces (Supplementary Materials and Methods, Supplementary Table S1). Moreover, chronic stress tends to increase rates of relaxation or oscillator rigidity (increased Floquet exponents) at a given level of adrenal sensitivity (k_p3_) since the value of the regression coefficient (b_3_), which determines the dependence of the Floquet exponent on adrenal sensitivity increases with increasing levels of chronic stress. The increase in rates of relaxation are correlated with a decrease in the domain of entrainment as mentioned above. Therefore, we suggest that while the simulated allostatic adaptation is associated with increased robustness to transient perturbation (inferred from the increased rates of relaxation), these changes are also indicative of a loss of the flexibility of the system to readjust to changes in environmental stimuli.

### Response to transient and permanent changes in the light-dark schedule

**Figure 4:**
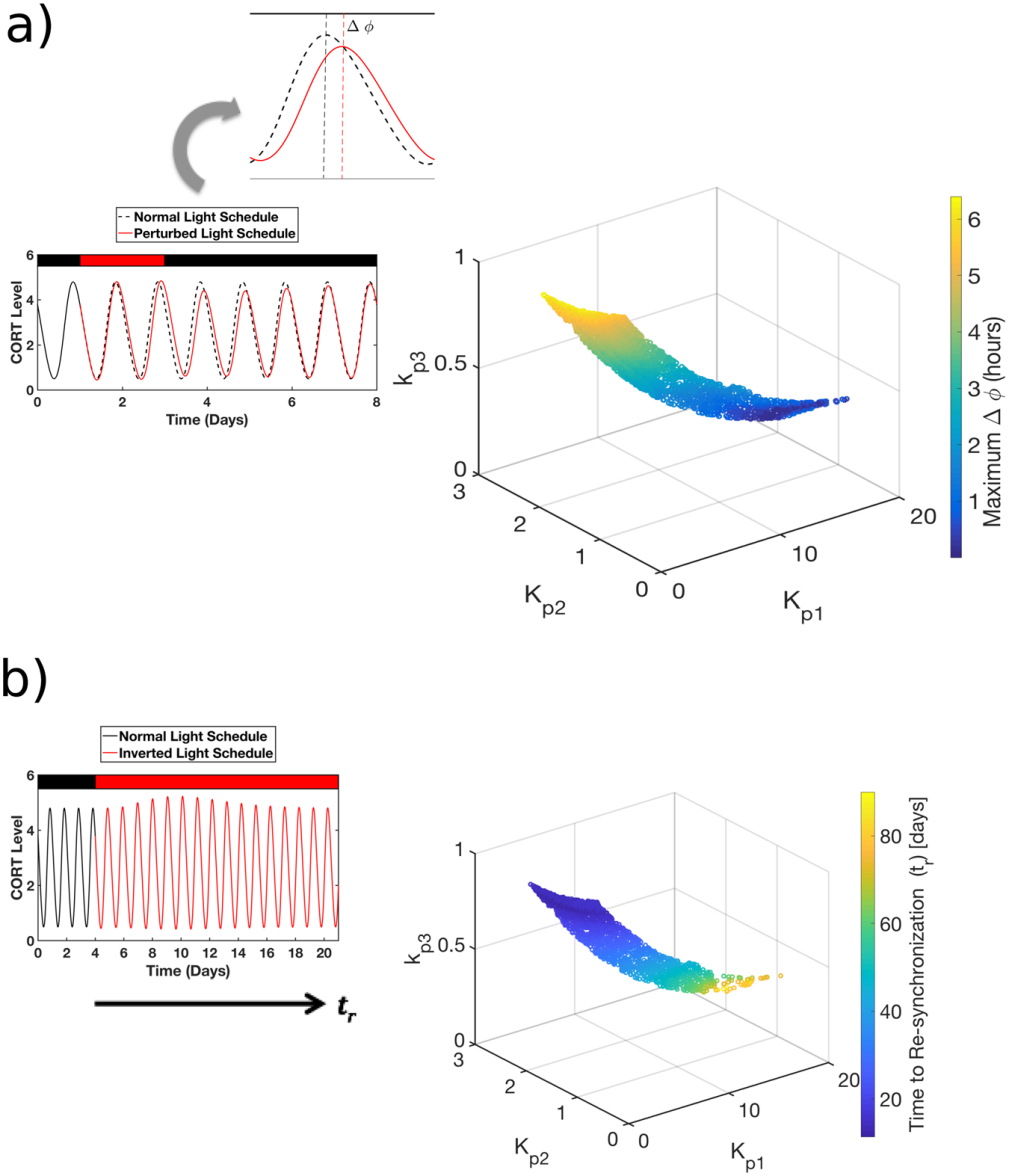
The response of the HPA axis to two physiologically relevant perturbations in the light/dark schedule was determined. (**a**) The maximal phase shift in the CORT rhythm was determined upon a transient inversion in the light/dark schedule lasting for 96 hours for the nominal surface. The maximal phase shift decreases with decreasing adrenal sensitivity (k_p3_). This implies that simulated individuals with lower adrenal sensitivity are more robust to transient perturbations in the light/dark cycle, such as those occurring during rapidly-rotating shift-work. (**b**) The nominal system was subjected to an abrupt inversion of the light-dark schedule. The color depicts the time required to resynchronize to the new light-dark schedule after this abrupt change. This implies that simulated individuals with higher adrenal sensitivity adapt more easily to permanent changes in the light-dark schedule similar to those occurring during trans-meridian jet-travel and are thus less susceptible to jet lag.

Given that regulatory variability results in differences in the intrinsic entrainment characteristics of the HPA axis, we next sought understand implications of this variability on the response of the entrained system to perturbations in the *zeitgeber*. We specifically focus on physiologically relevant perturbations in the light-schedule, since photoperiod is the key environmental cue synchronizing HPA rhythms in our model. In this respect, understanding the system’s response to perturbations in the light schedule can provide improved insight into how regulatory diversity might influence an individual’s tolerance to shiftwork or jet lag^43^. We first tested the hypothesis that differences in the regulatory constitution result in a differential response of the HPA axis to a transient inversion of the light/dark cycle. Studies suggest that the maximal circadian phase-shift during shift work is an important parameter that might be predictive of tolerance to shift work schedules^52,53^. Therefore, we characterized the maximal phase-shift in the corticosterone circadian rhythm following a transient inversion of the light/dark cycle. The phase shifts produced by the model during this protocol are comparable to those that have been observed in the experimental literature; on the order of a few hours during (rapidly rotating) shift work^53–56^. Our model predicts that the maximal phase-shift is greater for subjects with greater adrenal sensitivity and hypothalamic negative feedback (Fig. 4a). Moreover, the narrower the domain of entrainment (as quantified earlier via a smaller Arnold tongue) the more resilient to (corticosterone) phase changes the system is. Following this, we sought to determine how the underlying regulatory diversity influences the time required for the system to re-synchronize following a permanent shift in the light-dark schedule. The time to re-synchronization is considered to be an important parameter with implications for understanding factors determining tolerance to circadian disruption during jet lag^57^. Similar to the maximal phase perturbation following a transient perturbation in light-schedule, we find a parametric dependence of the time required for re-synchronization to the new light-dark schedule. Subjects exhibiting the maximal phase perturbation in response to a transient change in light/dark schedule also exhibit the shortest time to resynchronization upon a permanent change in light/dark schedule (Fig. 4b). In other words, subjects who were more susceptible to disruption were the fastest to adopt the new photoperiod schedule. Thus, our model simulations predict that simulated individuals with the greatest adrenal sensitivity and hypothalamic negative feedback take the least time for adaptation. Our results therefore highlight an interesting trade-off in that individuals that fare better in accommodating transient changes in photoperiod, (shift-work) are not able to adapt as swiftly to permanent changes in photoperiod (jet lag).

## Discussion

In this work we computationally characterize functional trade-offs inherent to the HPA axis. In doing so, we characterized diversity in the regulatory features of the HPA axis in both the homeostatic and chronically stressed states and their implications for understanding the effects of allostatic adaptation.

**Figure 5:**
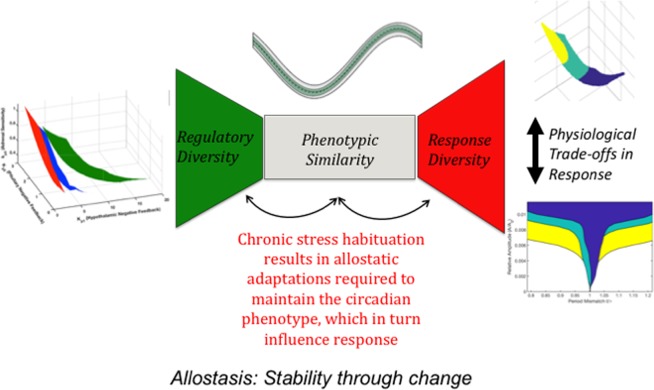
The variability in parametric surfaces is indicative of diverse individualized regulatory strategies through which the host can maintain circadian glucocorticoid rhythms, a critical physiological phenotype, within narrow homeostatic bounds. Furthermore, the underlying regulatory diversity results in flexibility in the response characteristics of the HPA axis, specifically its stress-responsive and entrainment properties. These features are suggestive of bow-tie architectures found in many complex physiological systems. Moreover, in order to conserve the circadian phenotype the system adjusts compensatory regulatory processes in a systematic manner that results in the existence of trade-offs between the stress-responsive and time-keeping functions of the HPA axis. Finally, allostatic habituation to chronic stress results in specific regulatory adaptations that alter the systems acute stress response and entrainment characteristics, indicative of the physiological cost (accumulation of allostatic load) associated with adaptation.

We hypothesize that regulatory diversity is necessary for the HPA axis to maintain homeostatic levels of its critical phenotype (i.e., tight operational glucocorticoid bounds). However, stress-induced allostatic maintenance of homeostasis, enabled through regulatory diversity, comes at a cost as the system, once adapted, needs to trade adaptation for responsiveness (Fig. 5). This conceptual framework is reminiscent of the “bow-tie” architectures suggested to underline the function of a multitude of physiological systems in their attempt to conserve functionality in the presence of operational variability^58^, thus, exemplifying trade-offs between numerous competing requirements. We suggest that an improved understanding of the dynamics of such architectures in the context of the HPA axis can provide new insights in to its functioning in both health and disease.

The circadian rhythmicity of endogenous glucocorticoids is partially indicative of the master circadian clock, located in the SCN, and has important implications for the homeostatic signaling of many physiological systems^59^. Our results suggest that despite substantial regulatory diversity, the HPA axis must adjust compensatory processes to maintain homeostatic levels of the GCs. We hypothesized that the existence of these diverse regulatory strategies to conserve the circadian phenotype will have significant consequences for the critical operational characteristics of the HPA axis, specifically its stress response and entrainment behavior.

In characterizing the entrainment properties of the system, we determined a) its domain of entrainment (as depicted by the Arnold tongues) as a metric of how flexibly the system is entrained to changes in the environmental zeitgeber (light/dark schedule) and b) the behavior of the system to physiologically relevant perturbations in the light/dark schedule. We conclude that regulatory structures with intrinsically wider domains of entrainment (ease of adaptation) exhibit greater sensitivity to abrupt but transient (acute) perturbations in the environmental zeitgeber. On the other hand, regulatory strategies exhibiting the largest phase disruptions in response to transient photoperiod perturbations are associated with improved abilities to re-synchronize following a permanent inversion of the light/dark schedule. These results are consistent with experimental findings, which have shown that oscillatory systems with narrow entrainment ranges generally respond with smaller phase changes in response to zeitgber stimuli^60,61^. Moreover, a number of investigators have shown that the range of entrainment of an oscillator is related to its phase response curve (PRC), a commonly used and powerful analytic tool for the characterization of circadian systems. In general, both theoretical and experimental studies show that a PRC with a smaller amplitude is associated with a narrow entrainment range, where the range of entrainer periods that an oscillator can adopt is indicated by the maximum and minimum values of the PRC^62,63^.

Our results highlight an important trade-off for all circadian systems between the ability to be a precise time-keeping mechanism *versus* the ability to be easily entrained to a rhythmic environmental stimulus. Since, the HPA axis conveys circadian information from the central SCN to peripheral tissues such a trade-off between entrainability and robust time-keeping functions is particularly important to consider^64,65^. These dynamics are of relevance towards understanding individual differences in circadian disruption occurring, for instance, during jet lag and shift-work. Recent evidence from adrenalectomy studies suggests that robust GC rhythmicity might buffer the phase of peripheral clocks against extrinsic noise. Moreover, Kiessling *et al*. used a mouse-model of jet-lag to show that manipulation of the GC rhythm could both quicken as well as slow-down the adaptation of activity cycles to changes in the light-dark schedule^57^. Experimentally re-synchronization of CORT rhythms after a substantial shift in the light/dark schedule has been reported to occur at about a day per time-zone shift in the schedule^7^. A substantial proportion of the simulated individuals in our virtual population have re-synchronization times comparable to experimental results despite the strict criterion for re-entrainment (phase difference between consecutive peaks less than 3 minutes). In the context of adaptation to jet lag it might beneficial for the circadian system to quickly resynchronize to a (permanent) change in light schedule, thus preventing prolonged periods of internal desynchronization with the external environment^57^. On the other hand, studies on shift work have found that individuals exhibiting a high amplitude circadian rhythm and a slow adjustment in the phase of the rhythm during the night-shift schedule were more tolerant to shift-work in a rapidly rotating shift system, reporting medical complaints at a lower frequency in comparison to individuals less resistant to shift-work^52,53^. Therefore, our model predicts qualitatively that individuals with robust endogenous glucocorticoid rhythmicity will be more tolerant to rapid shift-work rotations, while those with a wide domain of entrainment will be able to flexibly adapt to changes in zeitgeber characteristics. Interestingly, a recent study showed that in comparison to the mammary carcinoma-susceptible Fisher 344 rat strain, the resistant Copenhagen rat strain exhibited minimal phase disruption, to changes in the light/dark schedule that simulated shift-work^66^.

In addition to its synchronizing purpose, the HPA axis functions as the primary humoral stress response mechanism, with a flexible response to stressful external stimuli being essential for host fitness^6,67^. We find that individuals with higher adrenal sensitivity exhibit a more robust acute stress response. Moreover, given that the system adjusts compensatory regulatory processes in order to maintain the critical homeostatic circadian phenotype, we hypothesized that this might result in a systematic interdependent variation of the response characteristics. Therefore, we assessed whether the regulatory mechanisms of the HPA axis vary in a manner that couples the stress-responsive and entrainment properties of the HPA axis. Our results suggest that higher adrenal sensitivity and negative feedback makes the HPA easier to entrain. Thus, we predict that individuals with high adrenal sensitivity, and hence a more robust acute stress response, will more fluently adapt to jet lag, but will be more susceptible to circadian desynchrony due rapidly-rotating shift-work. Therefore, our model highlights the existence of a critical trade-off between the two primary physiological functions of the HPA axis: its ability to function as a robust timekeeper, resilient to fluctuations in an environment zeitgeber *versus* its ability to respond to environmental stressors. It is suggested that the optimal strategy between the stress responsiveness and entrainability on one hand, and robustness of rhythmicity will depend on the differences in individual life history and exposure to variable natural habitats. We hypothesize that the regulatory diversity might be representative of phenotypic plasticity where an individual organism is capable of modulating its phenotypic state in response to variations in its perceived environment so as to minimize transient accumulations of allostatic load^68^. Thus, adopting a robust phenotype might be beneficial to prevent over-activation in a highly variable natural habitat with frequent exposure to stressors, while adopting a more responsive phenotype might be advantageous in a relatively predictable natural habitat^69^. Therefore, the existence of diverse regulatory schemes might be reflective of multiple evolutionarily stable survival strategies in a context specific manner^46^.

Our results indicate that the accumulation of allostatic load, due to chronic stress, results in a substantial increase in the strength of hypothalamic negative feedback and adrenal sensitivity, accompanied by a reduction in regulatory flexibility (Fig. 2b). Interestingly, such an increase in adrenal sensitivity is in agreement with experimental findings, where subjects with post-traumatic stress disorder (PTSD) have been found to exhibit increased cortisol secretion in response to ACTH stimulation^70^. We suggest that the chronic stress-induced decrease in the variability of key regulatory features of the HPA axis results in a loss of flexibility of the system, which in turn renders the host more susceptible to subsequent environmental stressors. Natural variability in regulatory parameters is known to contribute to organismal fitness by facilitating effective adaptation to locally varying environments^71,72^. A number of studies suggest that natural allelic variation within the same population contributes to phenotypic variation that is thought to enable adaptive fine-tuning of the phenotype to environmental variation^72,73^. Deletion of the neuropeptide Y gene (NPY), which is involved in a protective response to stress, resulted in a decrease in inter-individual variability in clock period, a shortening of the clock period, and a slower adaptive response to abrupt changes in the photoperiod^74^. It has been hypothesized that maintenance of regulatory plasticity enables flexible adaptability in response to changing environmental conditions, with a loss in plasticity associated with reduced stress resilience and negative outcomes^68,75^.

Our model predicts that allostatic adaptations to a habituating chronic stressor result in an alteration of the acute stress response of the system. Interestingly, following habituation, the system exhibits a strong time-of-day dependence of the response to a subsequent acute stressor, with maximal response around the middle of the active phase. Notably, a number of studies have shown that habituation to prior chronic stress results in increased sensitivity upon subsequent exposure novel stressors^76–78^. This phenotype of chronic stress sensitization might be associated with negative outcomes, due to an increased risk of overexposure to GCs. Moreover, we predict that greater adrenal sensitivity leads to a greater sensitization of the acute stress response, and thus might be more likely to lead to a maladaptive response upon exposure to chronic stress. We hypothesize that this might have important implications for understanding individual differences in susceptibility to chronic stress disorders^79^.

Upon habituation to the chronic stress regimen we find that for the same level of adrenal sensitivity, the domain of entrainment becomes significantly narrower for increasing levels of chronic stress exposure, and thus the system loses is entrainability characteristics. Theoretical analyses^44^ predict this decrease in the domain of entrainment to be accompanied by a general increase in the robustness of oscillation to transient perturbation (as inferred from the increased rates of relaxation or Floquet exponents). From the viewpoint of a faithful timekeeper such an allostatic adaptation might seem beneficial by making the system a more rigid circadian clock. However, on the other hand, this also implies that habituation to chronic stress requires regulatory adaptations, which make it more difficult for the system to synchronize to diurnally varying environmental signals. Such a phenomenon of lack of entrainment of the internal circadian clock to external zeitgebers corresponds to non-24-hour sleep-wake syndrome, which has been observed in both sighted and blind patients^80,81^. Furthermore, this has also been observed in patients of schizophrenia^82^, and in subjects exposed to severe psychological stress^83^.

The mathematical model of the HPA axis we use in this study has a number of limitations. Importantly, in this work we focus on only the circadian rhythms of the HPA axis. However, it is well known that the mediators of the HPA axis additionally exhibit prominent ultradian rhythmicity, with a pulsatile frequency on the order of about one pulse per hour^84^. Moreover, there is disagreement about whether the ultradian and circadian rhythms of the mediators of the HPA axis are endogenous in nature, with recent evidence suggesting that the presence of circadian glucocorticoid rhythms after disconnection from the SCN being due to the peripheral adrenal clock^7^. Glucocorticoids have also been shown to have complex feedback effects on both peripheral clocks (including the adrenal peripheral clock) and central circadian clocks, thus adding further complexity to the circadian dynamics of glucocorticoids^85,86^. Nonetheless, it has been shown that many of the essential properties of glucocorticoid rhythms such as the observed temporal dependence of the stress response, amplitude behavior and entrainment properties can be explained using limit cycle oscillators as model systems^34,35,87,88^. In this sense, we suggest that many of the features of our simulation results might still be preserved even after separately accounting for these influences. Therefore, while our current model does not explicitly describe the disparate and complex influences of the SCN, the peripheral circadian clock, and multiple time-scales on glucocorticoid dynamics, we envision future modifications of the model that gradually incorporate these features.

## Conclusion

In summary, our model simulations indicate that complex physiological signaling systems such as the HPA axis can be viewed as having multiple, simultaneously competing functional objectives, which necessitate the existence of trade-offs between them. We predict the existence of individualized regulatory strategies, as represented by the parametric surfaces, that enable the system to conserve a basal circadian phenotype while also allowing for adequate flexibility in the stress-responsive and entrainment properties of the HPA axis. Our model predicts that this regulatory variability inextricably links the sensitivity of the HPA axis to external stressors with its ability to integrate and be entrained by environmental cues such as light and temperature. We hypothesize that individuals with high adrenal sensitivity, and hence greater stress-responsiveness, are also more easily entrained by, and more sensitive to zeitgeber stimuli. Thus, our results predict the existence of a trade-off between the stress-responsiveness of the HPA axis and its ability to be a faithful time-keeping mechanism that is robust to noise in environmental conditions. Moreover, we specifically highlight allostatic regulatory adaptations upon chronic stress habituation that influence the stress-responsiveness and entrainment properties of the HPA axis. Our models suggest that by allostatically habituating to chronic stress in a way that homeostatic glucocorticoid rhythms are maintained, the system is forced to compromise either its entrainment characteristics (adaptation) or its responsiveness to external stimuli, or in many cases both. In doing so, we further determine individual differences in the susceptibility to allostatic load accumulation. Specifically, we predict that individuals with greater adrenal sensitivity, exhibit a greater sensitization of the acute stress response upon allostatic habituation to a chronic stressor, and thus might be more susceptible to the incidence of chronic stress disorders. An improved understanding of the regulatory dynamics of the HPA axis can provide new insights into its functioning in both health and disease.

## Supplementary information

Supplementary Materials and Methods

## Data Availability

Additional data and materials generated in the study can be found in a public GitHub repository accessible at https://github.com/AndroulakisGrp/RR_Allostasis_HPA.
