# Supplementary material for: Allostatic adaptation and personalized physiological trade-offs in the circadian regulation of the HPA axis: A mathematical modeling approach
Source: Sci Rep. 2019 Aug 1;9:11212. doi: 10.1038/s41598-019-47605-7 (PMC6671996; doi:10.1038/s41598-019-47605-7)
Supplement: Supplementary file 1 — Supplementary Materials and Methods [file 41598_2019_47605_MOESM1_ESM.docx]

# **Allostatic adaptation and personalized physiological trade-offs in the circadian regulation of the HPA axis: A mathematical modeling approach**

Rohit Rao^1^ and Ioannis P. Androulakis^1, 2*^

^1^Department of Chemical and Biochemical Engineering, Rutgers, the State University of New Jersey, Piscataway,

^2^Department of Biomedical Engineering, Rutgers, the State University of New Jersey, Piscataway, NJ, 08854, USA^*^

*Indicates Corresponding Author

*Correspondence to [yannis@soe.rutgers.edu](mailto:yannis@soe.rutgers.edu)

# **Supplementary Methods**

## **Sampling Approach to Calibrate the Model for Experimental CORT Rhythms**

A sampling method based on Sobol sequences was used to construct the sample space representative of the homeostatic regulatory variability of the system, ^1^. Sobol sequences are an example of quasi-random low-discrepancy sequences, used to produce highly uniform samples of a unit hypercube. The algorithms generating Sobol sequences form successively finer partitions of the unit interval using a base of 2 and subsequently, reorder the generated coordinates in each dimension. The sampling procedure was implemented in MATLAB 2017a. The MATLAB commands sobolset and net were used to generate an initial quasi-random Sobol point set ^2,3^. A random linear scrambling algorithm combined with a random digital shift was applied to the initial Sobol point-set to generate the final sample space used for our sampling protocol in order to decrease the probability of undesired correlations in the initial segments of sequence ^4,5^.

The parameters that account for the GR pharmacodynamics were obtained from previous work by Ramakrishnan et al. ^6^ and were fixed. We, therefore, sampled remaining 11 parameters specific to the equations describing the dynamics of the CRH, ACTH and CORT. We used the parameter values from our previous work ^7^ as a seed parameter set and varied them within ±50%, to generate a set of 50,000 Sobol samples. We first identify a representative set of parameter values, that is able to most closely approximate the mean cosinor parameters of the experimentally obtained CORT profiles in female rats from Atkinson and Wadell^8^. Subsequently, we sampled only the parameters of interest, $K_{p1}$, $K_{p2}$ and $k_{p3}$ [Supplementary Table S2] such that that the circadian parameters of the simulated profiles lie within ±1 standard deviation of the experimentally obtained circadian parameters for the CORT profiles in ^8^ from the cosinor rhythmometry analysis. Further details regarding the sampling procedure are also presented in our previous work ^9^.

**Determining Influence of Individual Variability and Chronic Stress on the Flexibility of Entrainment**: The Arnold Tongue was calculated by entraining the system of equations to a sinusoidal zeitgeber of the form $F=X\left( 1+sin\left( \frac{2\pi}{T} \right) \right)$, here $X$ is the amplitude of sinusoidal zeitgeber and is indicative of the zeitgeber strength, and T is the period of the zeitgeber.

|  | $\frac{dCRH}{dt}=\frac{k_{p1}.K_{p1}}{K_{p1}+DR(N)}-V_{d1}.\frac{CRH}{K_{d1}+CRH}+F$ | Eq. S1 |
| --- | --- | --- |

The sinusoidal zeitgeber was coupled to the system through additive forcing as shown in Equation S1. For each parameter set, the zeitgeber strength was calculated relative to intrinsic amplitude A of the system, calculated in the absence of the influence of any external zeitgebers. For each value of the zeitgeber strength, the period of the zeitgeber was varied between ± 20% of the intrinsic period of the unentrained oscillator. The system was considered entrained if the phase difference was less than 3 minutes between every pair of consecutive peaks in a span of 8 consecutive peaks.

**Determining the Influence of Individual Variability and Chronic Stress on the Robustness of HPA Axis Oscillations**: The dynamics of the periodic oscillator with period T, can be represented by $\frac{d\boldsymbol{y}}{dt}=\boldsymbol{g}\left( \boldsymbol{y} \right)$. The introduction of external signals results in a perturbation, $\boldsymbol{\eta=y-}\boldsymbol{y}_{\boldsymbol{0}}$. around the stable solution $\boldsymbol{y}_{\boldsymbol{0}}$. For small deviations around the stable orbit, the dynamics of the perturbation can by given by $\frac{d\boldsymbol{\eta}}{dt}=D\boldsymbol{g}\left( \boldsymbol{y}_{\boldsymbol{0}}\left( \boldsymbol{t} \right) \right)\boldsymbol{\eta}(t)$. The general solution for perturbation takes the form $\boldsymbol{\eta}\left( t \right)=\sum_{i=1}^{N} c_{i}\exp\left( \mu_{i}t \right)p_{i}(t)$ where, $c_{i}$ are coefficients determined by the initial conditions, $p_{i}(t)$ are functions with period T, and $\mu_{i}$ are the Floquet exponents. The Floquet exponents are calculated by numerically integrating the matrix differential equation from t=0 to t=T, with initial conditions, $\boldsymbol{\eta}\left( t=0 \right)=\boldsymbol{I}$, where I is the identity matrix. The Floquet multipliers, $\rho_{i}$ are obtained as the eigenvalues of $\boldsymbol{\eta}\left( t=T \right)$. The Floquet exponents are related to the Floquet multipliers as follows: $\rho_{i}=\exp\left( \mu_{i}t \right)$. The Jacobian for the perturbation dynamics ($\frac{d\boldsymbol{\eta}}{dt}$) was evaluated using the in-built MATLAB function *numjac*, and the matrix differential equation was integrated numerically using MATLAB’s *ode45* routine.

**Multiple Linear Regression Analysis**

Multilinear regression analysis was performed to determine the dependence of the leading Floquet exponents on the three parameters of interest (${k_{p3}, K}_{p1}, K_{p2}$) in both the nominal and the two chronically stressed conditions considered. The general form of the regression equation is shown in Equation S2, where $\hat{\boldsymbol{y}}$ represents the leading Floquet exponent, $b_{0}$ represents the intercept, and $b_{1}, b_{2,}$and $b_{3}$ represent the weights determining the influence of the parameters of interest, respectively. The regression analysis was performed using MATLABs regress routine.

|  | $\hat{\boldsymbol{y}}= b_{0}+b_{1}.\boldsymbol{K}_{\boldsymbol{p}\boldsymbol{1}}+b_{2}.\boldsymbol{K}_{\boldsymbol{p}\boldsymbol{2}}+b_{3}\boldsymbol{.k}_{\boldsymbol{p}\boldsymbol{3}}$ | Eq. S2 |
| --- | --- | --- |

# **Supplementary Figures**


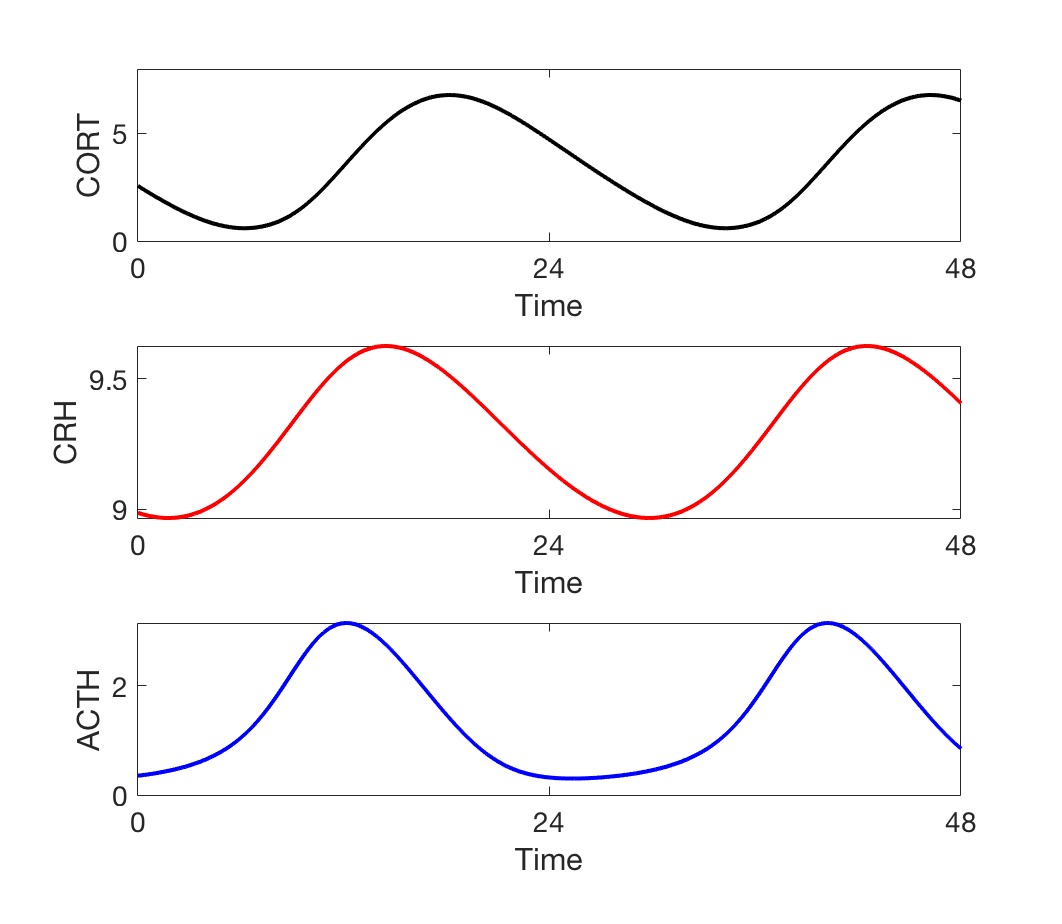


Figure S1: Representative profiles for CORT, CRH and ACTH for a sample point in the nominal parametric subspace.


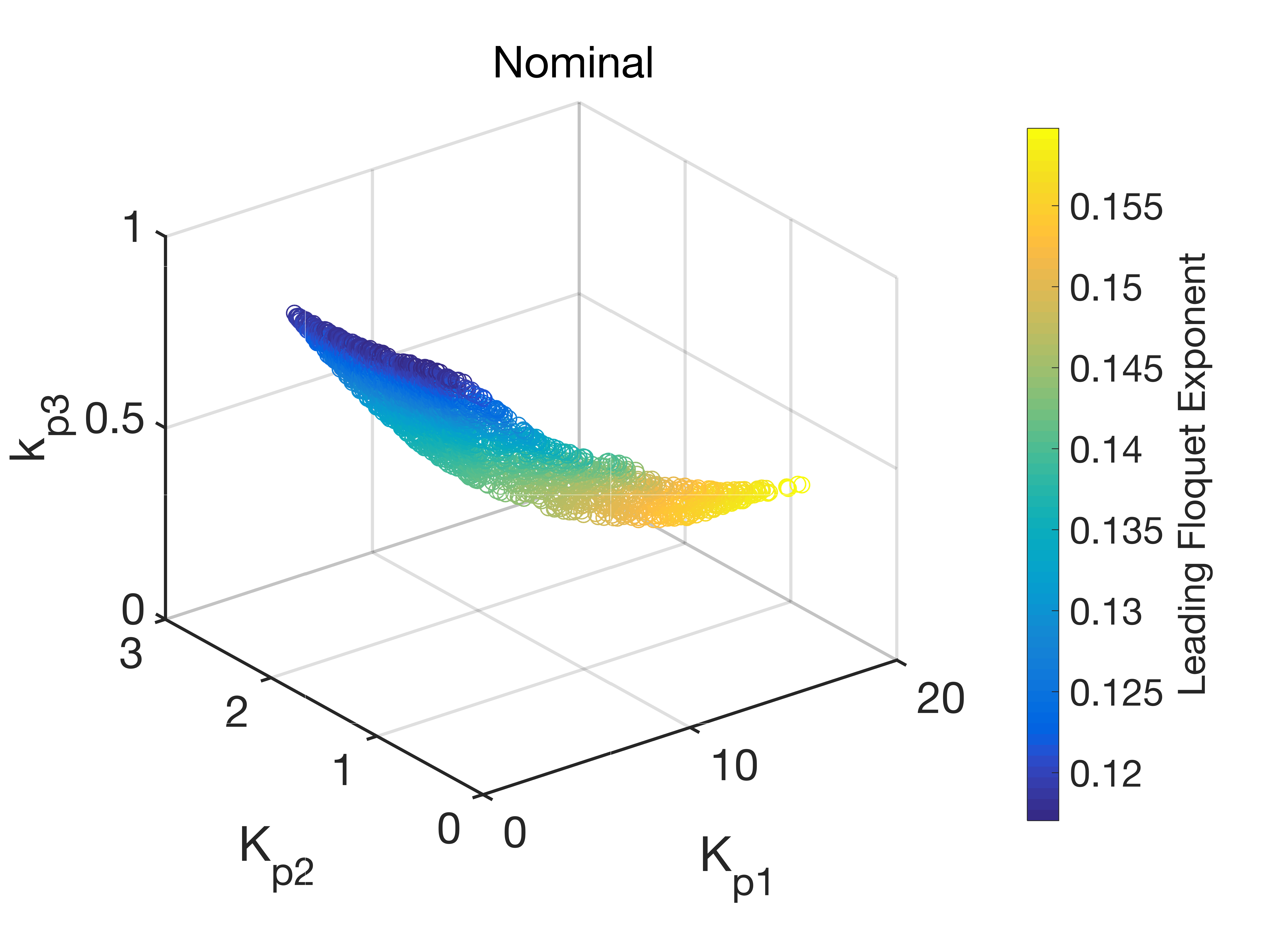


Figure S2: The Floquet exponent, indicative of the stability of the system to amplitude perturbation or in other words the robustness of its oscillatory characteristics. The Floquet exponent increases with decreasing adrenal sensitivity, sensitivity for the nominal condition with no chronic stress. This implies that individuals with higher adrenal sensitivity are less stable to amplitude perturbation. The color denotes the Floquet exponent of the unentrained system, with blue denoting the minimum Floquet exponent, while yellow denoting the maximum Floquet exponent.


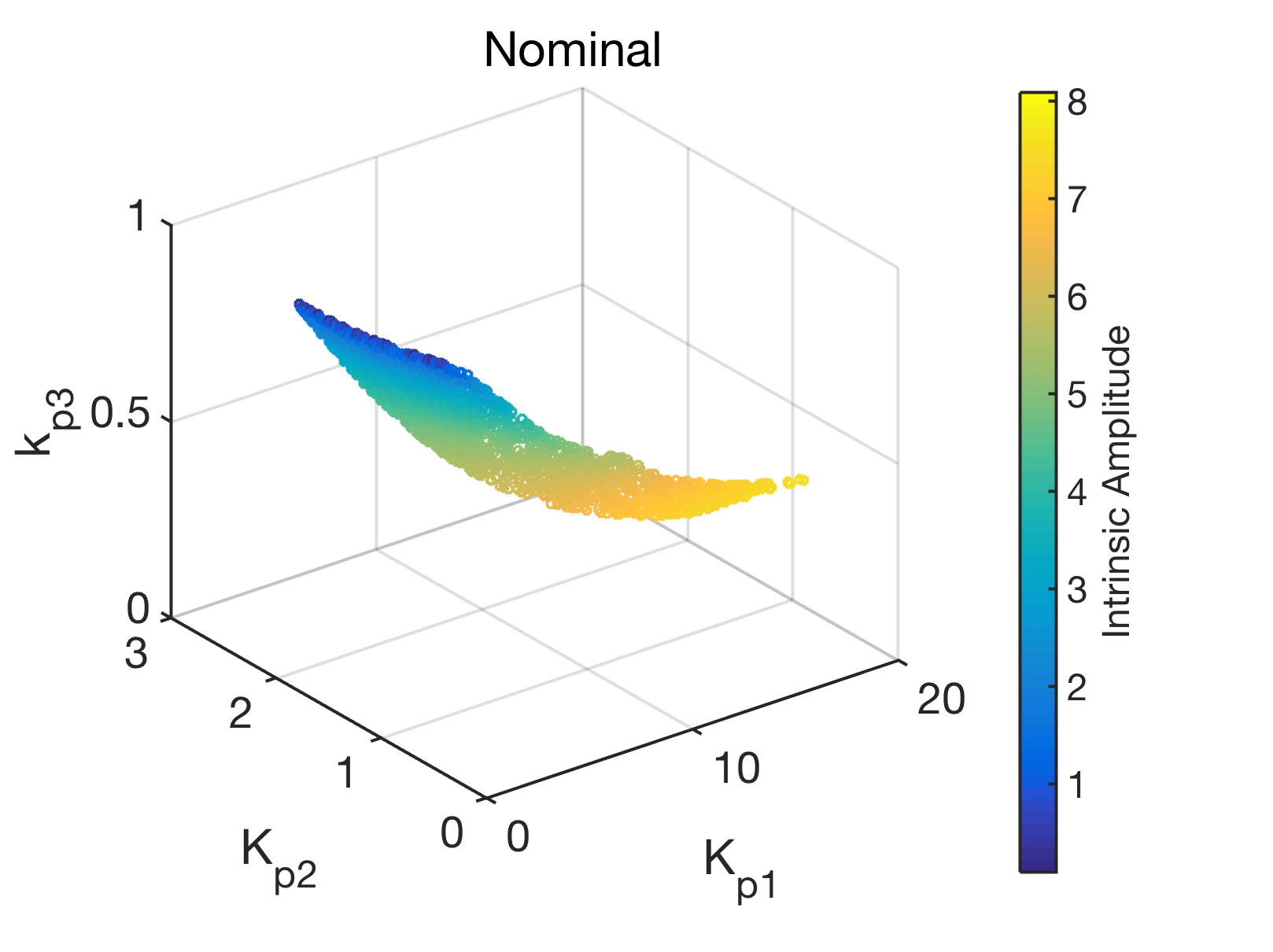


Figure S3: The intrinsic amplitude of the unentrained system increases with decreasing levels of adrenal sensitivity sensitivity. The color denotes the intrinsic amplitude of the unentrained system, with blue denoting the minimum amplitude, while yellow denoting the maximum amplitude.

# **Supplementary Tables**

Table S1: List of Parameters in the Model. Specific values for the parameters accounting for regulatory variability are provided on a public GitHub repository available at: <https://github.com/AndroulakisGrp/RR_Allostasis_HPA>

| Parameter | Value | Description |
| --- | --- | --- |
| $k_{p1}$ | 0.3819 µMh^-1^ | Estimated, zero order synthesis rate constant of CRH |
| $V_{d1}$ | 0.3492 µMh^-1^ | Estimated, first order rate constant for CRH degradation |
| $K_{d1}$ | 4.3875 µM | Estimated, Michaelis-Menten constant for CRH degradation |
| $k_{p2}$ | 0.4561 µMh^-1^ | Estimated, first order rate constant for synthesis of ACTH |
| $V_{d2}$ | 1.0015 µMh^-1^ | Estimated, first order rate constant for degradation of ACTH |
| $K_{d2}$ | 0.8488 µM | Estimated, Michaelis-Menten constant for ACTH degradation |
| $V_{d3}$ | 0.7245 µMh^-1^ | Estimated, first order rate constant for CORT degradation |
| $K_{d3}$ | 0.1807 µM | Estimated, Michaelis-Menten constant for CORT degradation |
| $GR(0)$ | 540.7 nmol L^−1^ mg protein^−1^ | Initial GR content, ^6^ |
| ${GR}_{mRNA}(0)$ | 25.8 fmolg^−1^ | Initial GR mRNA content, ^6^ |
| $k_{syn_{GRm}}$ | 2.9 fmolg^−1^ h^−1^ | Zero order rate constant for synthesis of GR mRNA, ^6^ |
| $r_{f}$ | 0.49 | GR recycle fraction from nucleus to cytoplasm, ^6^ |
| $k_{re}$ | 0.57 h^-1^ | Rate of GR recycling from nucleus to cytoplasm, ^6^ |
| $k_{on}$ | 0.00329 L nmol^−1^ h^−1^ | Second-order rate constant for CORT-GR binding, ^6^ |
| $k_{deg,GRm}$ | $k_{syn_{GRm}}/{GR}_{mRNA}(0)$ | First-order rate constant for degradation of GR mRNA, ^6^ |
| $k_{deg,GR}$ | 0.0572 h^-1^ | First order rate constant for degradation of GR, ^6^ |
| $k_{syn,GR}$ | $GR\left( 0 \right).k_{deg,GR}/ {GR}_{mRNA}(0)$ | First order rate constant for synthesis of GR, ^6^ |
| $k_{T}$ | 0.63 h^-1^ | Rate of GR translocation from cytoplasm to nucleus, ^6^ |
| $k_{imp}$ | 0.5 | Strength of ACTH impulse |
| $k_{stress.out}$ | 6.79 h^-1^ | Rate constant for clearance of stressor |
| $k_{s}$ | 40 | Strength of induction of CRH production by stressor |
| **Sampling Bounds for Parameters Accounting for Regulatory Variability** | | |
| **Parameter** | **Lower bound** | **Upper bound** |
| $K_{p1}$ (µM) | 0 | 6 |
| $K_{p2}$ (µM) | 0 | 20 |
| $k_{p3}$ (µMh^-1^) | 0 | 1.5 |

Table S2: Multi-linear regression analysis to assess the dependence of the leading Floquet exponent ($\hat{y}$) on the parameters representing the negative feedback ($K_{p1}$,$K_{p2}$) and adrenal sensitivity ($k_{p3}$) in our model.

| Regression Model: $\hat{\boldsymbol{y}}= b_{0}+b_{1}.\boldsymbol{K}_{\boldsymbol{p}\boldsymbol{1}}+b_{2}.\boldsymbol{K}_{\boldsymbol{p}\boldsymbol{2}}+b_{3}\boldsymbol{.k}_{\boldsymbol{p}\boldsymbol{3}}$ | | | | |
| --- | --- | --- | --- | --- |
| Condition | $b_{0}$ [95% CI] | $b_{1}$ [95% CI] | $b_{2}$ [95% CI] | $b_{3}$ [95% CI] |
| Nominal | 0.1897  [0.1892 0.1902] | 0.00106  [0.00103 0.00109] | 0.0115  [0.0112 0.0118] | -0.1311  [-0.1321 -0.1302] |
| Intermediate Stress | 0.1825  [0.1812 0.1837] | 0.0005  [0.0002 0.0008] | 0.0052  [0.0045 0.0060] | -0.0809  [-0.0830 -0.0787] |
| High Stress | 0.1783  [0.1764 0.1801] | -0.0009  [-0.0018 -0.00005] | 0.0014  [0.0002 0.0026] | -0.0565  [-0.0591 -0.0539] |

Table S3: Criteria for constructing the parametric subspace representative of homeostatic regulatory variability in the HPA axis.

| **Scaled Experimental Cosinor Parameters** | |
| --- | --- |
| $f\left( t \right)=M_{exp}+A_{exp}\cos\left( 2\pi\frac{\left( t-\theta_{exp} \right)}{24} \right)$ | |
| Feature | Female |
| Mean ($M_{exp})$ | 2.40 ± 0.14 |
| Amplitude ($A_{exp})$ | 2.2 ± 0.42 |
| Phase ($\theta_{exp})$ | 20.7 ± 0.9 |
| **Error Criteria for Simulated Cosinor Parameters** | |
| Mean ($M_{sim}$) | ${M_{exp}-\sigma_{M}\leq M}_{sim}\leq M_{exp}+ \sigma_{M}$ |
| Amplitude ($A_{sim})$ | ${A_{exp}-\sigma_{A}\leq A}_{sim}\leq A_{exp}+\sigma_{A}$ |
| Phase ($\theta_{sim})$ | ${\theta_{exp}-\sigma_{\theta}\leq\theta}_{sim}\leq\theta_{exp}+\sigma_{\theta}$ |

Table S4: Sample parameter sets on nominal surface used to form Arnold Tongues. Specific values for all sets of parameters accounting for regulatory variability are provided on a public GitHub repository available at: <https://github.com/AndroulakisGrp/RR_Allostasis_HPA>

| $K_{p1}$ (µM) | $K_{p2}$ (µM) | $k_{p3}$ (µMh^-1^) |
| --- | --- | --- |
| 11.524 | 0.8057 | 0.501 |
| 8.714 | 0.995 | 0.499 |
| 6.649 | 1.242 | 0.499 |
| 8.057 | 1.207 | 0.619 |
| 6.152 | 1.491 | 0.619 |
| 4.854 | 1.782 | 0.620 |
| 6.537 | 1.628 | 0.728 |
| 5.122 | 1.950 | 0.728 |
| 4.133 | 2.219 | 0.728 |

**References**

1 Antonov, I. A. & Saleev, V. An economic method of computing LP τ-sequences. *USSR Computational Mathematics and Mathematical Physics* **19**, 252-256 (1979).

2 Bratley, P. & Fox, B. L. Algorithm 659: Implementing Sobol's quasirandom sequence generator. *ACM Transactions on Mathematical Software (TOMS)* **14**, 88-100 (1988).

3 Joe, S. & Kuo, F. Y. Remark on algorithm 659: Implementing Sobol's quasirandom sequence generator. *ACM Transactions on Mathematical Software (TOMS)* **29**, 49-57 (2003).

4 Hong, H. S. & Hickernell, F. J. Algorithm 823: Implementing scrambled digital sequences. *ACM Transactions on Mathematical Software (TOMS)* **29**, 95-109 (2003).

5 Jiri & Matousek. On the L2-discrepancy for anchored boxes. *J. Complex.* **14**, 527-556, doi:10.1006/jcom.1998.0489 (1998).

6 Ramakrishnan, R., DuBois, D. C., Almon, R. R., Pyszczynski, N. A. & Jusko, W. J. Fifth-generation model for corticosteroid pharmacodynamics: application to steady-state receptor down-regulation and enzyme induction patterns during seven-day continuous infusion of methylprednisolone in rats. *J Pharmacokinet Pharmacodyn* **29**, 1-24 (2002).

7 Mavroudis, P. D., Corbett, S. A., Calvano, S. E. & Androulakis, I. P. Mathematical modeling of light-mediated HPA axis activity and downstream implications on the entrainment of peripheral clock genes. *Physiological Genomics* **46**, 766-778, doi:10.1152/physiolgenomics.00026.2014 (2014).

8 Atkinson, H. C. & Waddell, B. J. Circadian variation in basal plasma corticosterone and adrenocorticotropin in the rat: sexual dimorphism and changes across the estrous cycle. *Endocrinology* **138**, 3842-3848, doi:10.1210/endo.138.9.5395 (1997).

9 Rao, R. T. & Androulakis, I. P. Modeling the Sex Differences and Inter-individual Variability in the Activity of the Hypothalamic-Pituitary-Adrenal Axis. *Endocrinology*, doi:10.1210/en.2017-00544 (2017).
